# SMOTE-CD: SMOTE for compositional data

**DOI:** 10.1371/journal.pone.0287705

**Published:** 2023-06-29

**Authors:** Teo Nguyen, Kerrie Mengersen, Damien Sous, Benoit Liquet

**Affiliations:** 1 Laboratoire de Mathématiques et de leurs Applications, Université de Pau et des Pays de l’Adour, E2S UPPA, CNRS, Anglet, France; 2 School of Mathematics and Physical Sciences, Macquarie University, Sydney, NSW, Australia; 3 School of Mathematical Sciences, Queensland University of Technology, Brisbane, QLD, Australia; 4 Laboratoire des Sciences Pour l’ingénieur Appliquées à la Mécanique et au Génie Électrique, Université de Pau et des Pays de l’Adour, E2S UPPA, Anglet, France; 5 Mediterranean Institute of Oceanography, Université de Toulon, Aix Marseille Université, CNRS, IRD, La Garde, France; Jeonbuk National University, KOREA, REPUBLIC OF

## Abstract

Compositional data are a special kind of data, represented as a proportion carrying relative information. Although this type of data is widely spread, no solution exists to deal with the cases where the classes are not well balanced. After describing compositional data imbalance, this paper proposes an adaptation of the original Synthetic Minority Oversampling TEchnique (SMOTE) to deal with compositional data imbalance. The new approach, called SMOTE for Compositional Data (SMOTE-CD), generates synthetic examples by computing a linear combination of selected existing data points, using compositional data operations. The performance of the SMOTE-CD is tested with three different regressors (Gradient Boosting tree, Neural Networks, Dirichlet regressor) applied to two real datasets and to synthetic generated data, and the performance is evaluated using accuracy, cross-entropy, F1-score, R2 score and RMSE. The results show improvements across all metrics, but the impact of oversampling on performance varies depending on the model and the data. In some cases, oversampling may lead to a decrease in performance for the majority class. However, for the real data, the best performance across all models is achieved when oversampling is used. Notably, the F1-score is consistently increased with oversampling. Unlike the original technique, the performance is not improved when combining oversampling of the minority classes and undersampling of the majority class. The Python package *smote-cd* implements the method and is available online.

## Introduction

### Context

Over the past few years, data imbalance problems have been widely studied in classification tasks [1]. An imbalance distribution over the classes will often cause the models to prioritize their performance on the majority classes, at the expense of the minority ones. Different methods exist to deal with imbalanced datasets [2]: algorithm-level methods, where the algorithm reduces the bias by inducing a weight on the classes; data-level methods, where the data are modified to reach a more balanced state; and hybrid methods, combining both algorithm-level methods and data-level methods. Among data-level methods, Synthetic Minority Oversampling TEchnique (SMOTE) [3], with all its variations [4], is one of the most popular for classification problems. The SMOTE algorithm generates synthetic data points for a particular class by combining the features of two existing points belonging to the same class through linear interpolation.

Most algorithms designed to tackle class imbalance problems, such as SMOTE, are often limited to the classification tasks; for instance [5–9]. However, even though regression problems are also very common in real-life problems, only a few resampling strategies exist for regression tasks [10, 11].

In this paper, we address the special issue of dealing with an imbalanced dataset in regression problems in the case where the labels are compositional. Compositional data are data carrying relative information [12], presented as proportions or percentages, making them different from other types of data. Compositional data are encountered in various fields, including biology [13–15], chemistry [16, 17], ecology [18, 19], geology [20, 21], and social sciences [22–24], among others. However, the class imbalance problem in compositional data regression remains a major challenge in the development of effective models. Existing adaptations of SMOTE and other oversampling techniques have focused on addressing imbalanced datasets in single-label regression [25–28], multi-label classification [29, 30], or when the features are compositional data [31]. However, to the best of our knowledge, no oversampling technique exists for addressing the issue of class imbalance in multi-label regression problems with compositional labels. Therefore, we propose a new oversampling technique called SMOTE for Compositional Data (SMOTE-CD), specifically designed to address this particular situation.

Here, we will measure class imbalance by summing the values of the labels (probability values) for each class on the whole dataset, and summarizing it as a percentage. In that sense, in a perfectly balanced dataset, the percentage of the sum of each class would be 1/*K*, with *K* being the number of classes.

The proposed method is evaluated using five different performance metrics, including accuracy, cross-entropy, F1-score, R2 score, and RMSE, to three different models (Gradient Boosting tree, Neural Networks, Dirichlet regressor) on both simulated and real datasets. Since no other oversampling algorithm currently exists for compositional data, the evaluation of SMOTE-CD is limited to comparing its performance against the case where no oversampling technique is applied. The results show that the performance of the models is overall greater when applying SMOTE-CD, thus demonstrating the effectiveness of the proposed method. This is an important contribution to the field, as it provides a solution for dealing with compositional data imbalance, which has not been addressed before. The use of five different evaluation metrics, as well as the application of three different models to both simulated and real datasets, further strengthens the reliability and generalizability of the proposed method.

The entire paper is arranged as follows. The paper’s first section introduces the proposed method and the motivation example. Section 2 presents the compositional data and the SMOTE-CD algorithm. Section 3 presents the metrics, the simulation study and its results. Sections 4 and 5 present the result on the real datasets. Section 6 presents the discussion and conclusion.

### Motivation example: Maupiti island

#### Description of Maupiti island

The overall purpose of our research project is to develop an automated mapping tool able to provide a classification map from a given satellite image, with a particular focus on a coral reef-lagoon system. The test field site is the Maupiti island, the westernmost Leeward island of the Society archipelago, French Polynesia. The site has a size of approximately 8km by 8km. Maupiti data, that we use here, is just an example, but compositional data can be found, for instance, in health or chemistry fields.

**Fig 1:**
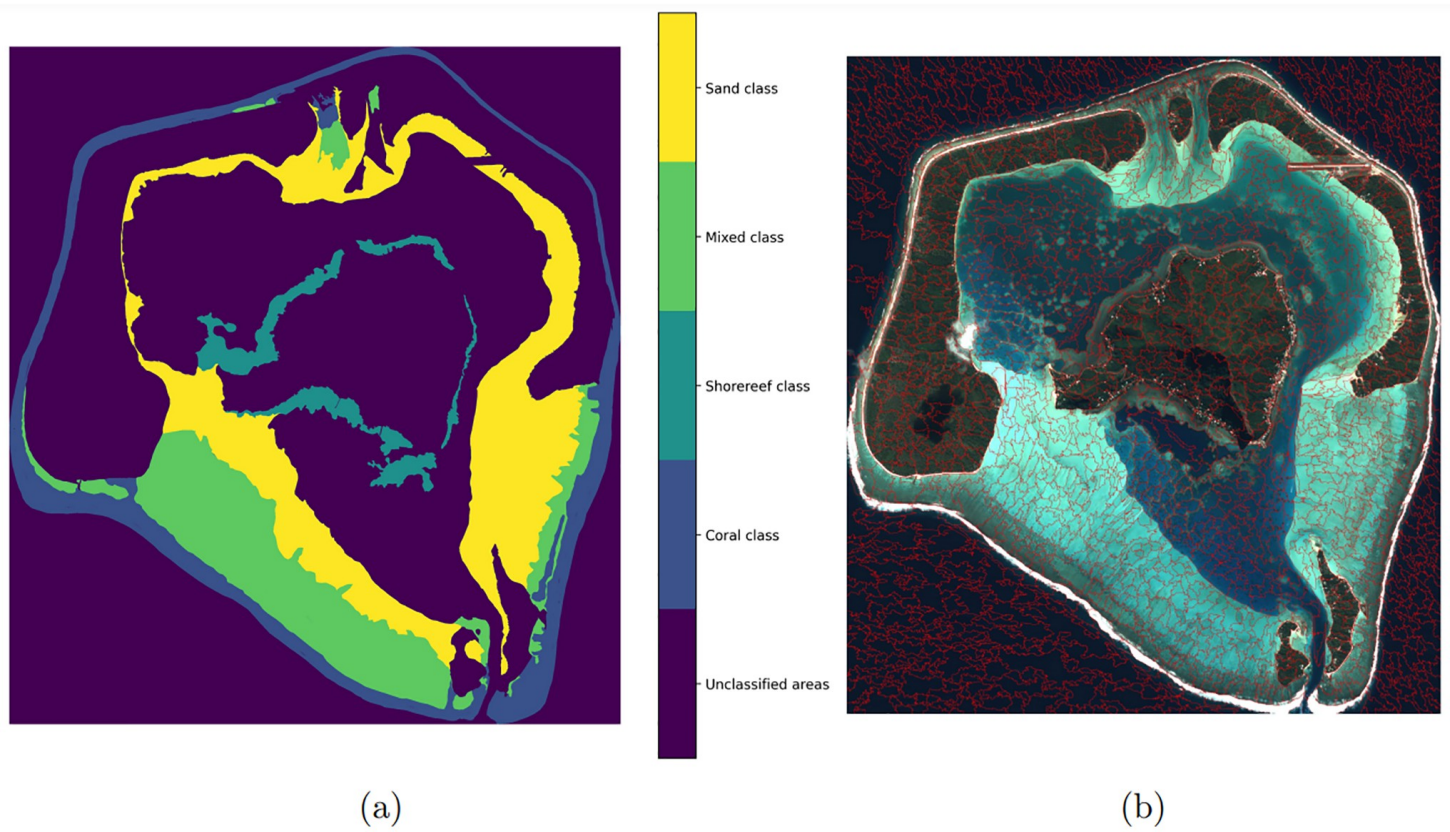
(a) Expert-based mapped image of Maupiti island and (b) Pleiades image of Maupiti island segmented with Felzenszwalb’s method.

An expert-based mapping of Maupiti island was used as a training dataset to develop the model. The satellite image used is a 4-band image captured on June, 14 2021 by the Pleiades satellite. The expert-based mapping of the image relies on the combination of several field observation campaigns [32] and direct examination of the satellite image. The present analysis focuses on the shallow regions of the lagoon, displaying more interpretable imaging. In the selected areas, four seabed type classes were established (Fig 1a):

- **Class 1: Coral**, marked by a overwhelming dominance of coral reef cover.
- **Class 2: Sand**, describing areas covered by detritic sand.
- **Class 3: Shorereef**, gathering shore reef and transitional shore reef.
- **Class 4: Mixed**, representing area covered by a combination of sand and coral.

#### Automatic mapping

To perform the automatic mapping, the image was first segmented using Felzenszwalb’s method [33], which gives Fig 1. For each segment, two different operations were applied:

- The four statistical moments (mean, variance, skewness, kurtosis) were computed on each band; these 16 values will be the features of the dataset.
- The percentage of pixels belonging to each class were computed, according to the expert-based classification; this results in a vector that sums up to 1 that will be the labels of the dataset.

To be able to map the satellite image, the idea was to train a regressor to retrieve, for each segment, the percentage of pixels belonging to each class (i.e., a vector of probabilities). As shown in Table 1, the data are not balanced: one of the class represents 49.5% of the dataset, while another one represents only 3.6%. To overcome this issue, we developed an oversampling technique in order to improve the performance of the regression model on this special kind of data.

**Table 1:** Percentage of the number of pixels of each class on Maupiti data, based on expert mapping.

Class	Class 1	Class 2	Class 3	Class 4
Percentage	0.117	0.040	0.482	0.361

## Materials and method

### Compositional data

Mathematically, we define a *D*-part compositional dataset as a vector $x=\left(x_{1},x_{2},\ldots ,x_{D}\right)\in R^{D}$ such that,

$$\begin{array}{c} \left\{ \begin{array}{c} x_{i}\geq 0,\;\forall \;i\in \left\{1,2,\ldots ,D\right\},\\ \sum_{i=1}^{D}x_{i}=1. \end{array}\right. \end{array}$$

A simplex *S*^*D*^ is defined as the ensemble of all the *D*-part compositional data, i.e.

$$\begin{array}{c} S^{D}=\{x=\left(x_{1},x_{2},...,x_{D}\right)\;|\;\forall i\in \left\{1,2,\ldots ,D\right\},x_{i}\geq 0;\sum_{i=1}^{D}x_{i}=1\}. \end{array}$$

The operations performed in *S*^*D*^ must be adapted to follow the properties of the simplex [12]. For instance, before performing the Euclidian operations, it is possible to first apply the centred log-ratio transform *clr*(⋅) to the data,

$$\begin{array}{cc} clr:S^{D} & \to R^{D}\\ \left(x_{1},\ldots ,x_{D}\right) & \mapsto (\text{log}\;(\frac{x_{1}}{g(x)}),\ldots ,\text{log}\;(\frac{x_{D}}{g(x)})). \end{array}$$

where the function *g*(⋅) is the geometric mean $g\left(x\right)={\left(\prod_{i=1}^{D}x_{i}\right)}^{\frac{1}{D}}$. The *clr*(⋅) function is only defined for vectors where none of the value is equal to 0. Several methods exist to overcome this issue [34], but in practice we just replace the 0 by a tiny value such as 10^−20^. The definition of the *clr*(⋅) function involves the existence of the inverse function *clr*^−1^(⋅), that turns to be the softmax function, defined for $z=\left(z_{1},\ldots ,z_{D}\right)\in R^{D}$ as

$$\begin{array}{c} \text{softmax}\left(z\right)=\frac{1}{\sum_{i=1}^{D}\;\text{exp}\;\left(z_{i}\right)}\cdot \left(\text{exp}\;\left(z_{1}\right),\ldots ,\;\text{exp}\;\left(z_{D}\right)\right). \end{array}$$

It is also possible to directly define operators on *S*^*D*^. Let *C* be the closure operator,

$$\begin{array}{c} \forall k\in N,C\left(x_{1},\ldots ,x_{k}\right)=\left(x_{1},\ldots ,x_{k}\right)/\left(x_{1}+\cdots +x_{k}\right). \end{array}$$

For two *D*-part compositions *x*, *y* ∈ *S*^*D*^, the perturbation *x* ⊕ *y* is defined by

$$\begin{array}{c} x⊕y=C(x_{1}y_{1},\ldots ,x_{D}y_{D}), \end{array}$$ (1)

and, given α∈R, the power transformed composition *α* ⊕ *x* is

$$\begin{array}{c} \alpha ⊗x=C(x_{1}^{\alpha },\ldots ,x_{D}^{\alpha }). \end{array}$$ (2)

### SMOTE for compositional data

In this section, we denote by *n* the number of samples in the dataset, *p* the number of features and *K* the number of classes. The matrix $X\in R^{n\times p}$ contains the *n* observations of the *p* features and $Y\in R^{n\times K}$ contains their labels. For any *i* ∈ {1, …, *n*} and *j* ∈ {1, …, *K*}, we denote by *y*_*i*,*j*_ the value of *Y* at row *i* and column *j*, and *y*_*i*,⋅_ = (*y*_*i*,1_, …, *y*_*i*,*K*_) the probability vector label of row *i*. Similarly, for *i* ∈ {1, …, *n*} and *j* ∈ {1, …, *p*}, *x*_*i*,*j*_ is the value of *X* at row *i* and column *j*, and *x*_*i*,⋅_ = (*x*_*i*,1_, …, *x*_*i*,*p*_). In order to simplify the notation, we define

$$\text{argmax}\left(y_{i,\cdot }\right)=\underset{j\in \{1,\ldots ,K\}}{\text{argmax}}\left(y_{i,j}\right),$$

which represents the majority class of a given label *y*_*i*,⋅_ ∈ [0, 1]^*K*^. We also define the sum vector $S\in R^{K}$ as the sum of the values for each class,

$$\begin{array}{c} S=(\sum_{i=1}^{n}y_{i,1},\sum_{i=1}^{n}y_{i,2},\ldots ,\sum_{i=1}^{n}y_{i,K}). \end{array}$$ (3)

The majority class of the dataset is thus defined as argmax(*S*), and the minority class as argmin(*S*).

**Fig 2:**
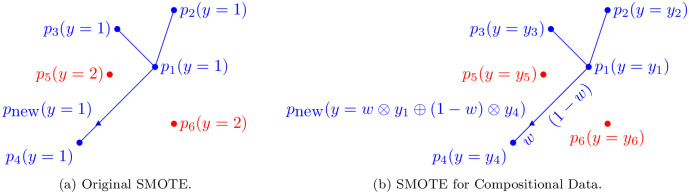
Difference between the original SMOTE algorithm and SMOTE-CD. The blue points are the points to oversample. (a) The points to oversample belong to the same class (here, class 1). (b) The points to oversample are the ones that have the same class as their majority class in their compositional vector label.

Before introducing the SMOTE-CD algorithm, let’s first summarize the idea behind the original SMOTE algorithm. As shown in Fig 2(a), the SMOTE algorithm creates a new point that belongs to class 1 (represented by blue points). To achieve this, the algorithm first selects a point at random (in this case, *p*_1_) and identifies its nearest neighbors (*p*_2_, *p*_3_, *p*_4_). Note that only neighbors with the same label as *p*_1_ (i.e., class 1) are considered, while points labeled as class 2 (represented by red points) are ignored. The algorithm then chooses one of these neighbors (*p*_4_) and creates a new point along the line that connects *p*_1_ and *p*_4_. The features of the new point are determined through a linear combination of the features of *p*_1_ and *p*_4_, and its label is assigned as 1. Algorithm 1 describes the SMOTE algorithm.

**Algorithm 1** Original SMOTE [3]

**Require**: $X\in R^{n\times p}$ the features.

**Require**: *Y* ∈ {1, …, *J*}^*n*^ the class label outputs.

**Require**: k∈N the number of neighbors to select for the *k*-Nearest Neighbors.

**Ensure**: Generated data $X_{\text{new}}\in R^{q\times p}$ and *Y*_new_ ∈ {1, …, *J*}^*q*^ with *q* the number of points created.

1: Denote by *S*_*j*_ the number of points labeled as class *j*.

2: M ← the majority class of dataset.

3: Initialize *X*_new_ and *Y*_new_ as empty matrices.

4: **for** every class *m* that needs to be oversampled **do**

5:  **while** *S*_*m*_ < *S*_*M*_ **do**

6:   Compute $D=\{i\;|\;y_{i}=m$}, the set of points labeled as class *m*.

7:   Randomly choose $r_{1}\in D$ and find the indices of its *k* nearest neighbors.

8:   Randomly choose an index *r*_2_ among these neighbors.

9:   $x^{\text{new}}\leftarrow w\times x_{r_{1,\cdot }}+(1-w)\times x_{r_{2,\cdot }}$ with *w* ∈ [0, 1] randomly drawn.

10:   *y*^*new*^ ← *m*.

11:   *S*_*m*_ ← *S*_*m*_ + 1.

12:   Append *x*^*new*^ to *X*_new_, append *y*^*new*^ to *Y*_new_.

13:  **end while**

14: **end for**

15: **return** *X*_new_, *Y*_new_

The SMOTE-CD algorithm keeps the main ideas from the original SMOTE: 1) select a point from the class to be oversampled, 2) select one of its *k*-Nearest Neighbors (k∈N specified by the user) and 3) create a synthetic point in-between those two points. Because of the label that is compositional, these three steps have to be adapted:

1. Select a point *r*_1_ whose majority class is *m*, where *m* is the minority class of the dataset.
2. Compute the *k*-Nearest Neighbors of *r*_1_ among the points that also have *m* as their majority class. Then select a point *r*_2_ in one of these *k* neighbors.
3. Randomly draw *w* ∈ [0, 1]. The features of the point to be created is a linear combination of the two points selected before, with *w* being the weight of *r*_2_ and (1 − *w*) the weight of *r*_1_. Similarly, the labels of the point to be created is a linear combination, but using the operators from Eqs (1) and (2).

Fig 2(b) depicts an example of how SMOTE-CD creates a new point. As we are dealing with compositional data label, every point *p*_*i*_ has a vector label *y*_*i*_. All the blue points are the points having the class *m* as the majority class of their label *y*_*i*_, where *m* is the minority class of the dataset. The algorithm computes the 3 nearest neighbors of *p*_1_ only considering the blue points, and then a point is created on the line between *p*_1_ and *p*_4_. The label of the new point is a linear combination of the labels *y*_1_ and *y*_4_ using the operations defined on the simplex (Eqs (1) and (2)).

Algorithm 2 describes the SMOTE-CD algorithm, using the same notation.

**Algorithm 2** SMOTE for compositional data

**Require**: $X\in R^{n\times p}$ the features.

**Require**: $Y\in R^{n\times K}$ the labels (compositional data).

**Require**: k∈N the number of neighbors to select for the *k*-Nearest Neighbors.

**Ensure**: Generated data *X*_new_ ∈ *R*^*q*×*p*^ and *Y*_new_ ∈ *R*^*q*×*K*^ with *q* the number of points created.

1: Compute the label sum vector $S\in R^{D}$ as defined in Eq (3).

2: M ← argmax(*S*), the majority class of dataset (hence *S*_*M*_ is the sum of the majority class).

3: Initialize *X*_new_ and *Y*_new_ as empty matrices.

4: **while** min(*S*) < *S*_*M*_ **do**

5:  *m* ← argmin(*S*), the minority class of dataset.

6:  Compute $D=\{i\;|\;\text{argmax}(y_{i,\cdot })=m\}$, the set of points whose majority class is *m*.

7:  Randomly choose an index $r_{1}\in D$.

8:  Find the indices of the *k* nearest neighbors of *r*_1_ in D, using the Euclidian distance on *X*.

9:  Randomly choose an index *r*_2_ among these indexes.

10:  Uniformly draw a number *w* ∈ [0, 1].

11:  $x^{\text{new}}\leftarrow w\times x_{r_{1,\cdot }}+(1-w)\times x_{r_{2,\cdot }}$.

12:  $y^{\text{new}}\leftarrow w⊗y_{r_{1,\cdot }}⊗(1-w)c⊗y_{r_{2,\cdot }}$.

13:  *S* ← *S* + *y*^*new*^.

14:  Append *x*^*new*^ to *X*_new_, append *y*^*new*^ to *Y*_new_.

15: **end while**

16: **return** *X*_new_, *Y*_new_

The step that creates the label of the new point (line 12) uses the definitions of Eqs (1) and (2). Nevertheless, it is also possible to create the label by using the Euclidian operations on the logratio transformed labels, and to apply the inverse transformation afterwards: $clr^{-1}(w\times clr(y_{r_{1,\cdot }})+(1-w)\times clr(y_{r_{2,\cdot }}))$. Although the label could be created by directly performing Euclidian operations on the compositional label, however this would be mathematically irrelevant because it would not respect the rules of compositional data analysis [35].

The proof of convergence holds in the fact that, at each iteration, the increase of the major class of *S* is smaller that the increase of its minor one, causing the sum of the minor class to converge to the sum of the major one. In other words, we have to be assured that, at each iteration, $y_{m}^{\text{new}}>y_{M}^{\text{new}}$, with *m* (resp. *M*) the minority (resp. majority) class of the dataset.

This is straightforward by noticing that the two indices *r*_1_ and *r*_2_ used for generating a new point are chosen in D={i| argmax(*y*_*i*,⋅_) = *m*}:

$$\begin{array}{cc} r_{1},r_{2}\in D & \Rightarrow \{\begin{array}{c} y_{r_{1},m}>y_{r_{1},M}\\ y_{r_{2},m}>y_{r_{2},M} \end{array}\\ & \Rightarrow \{\begin{array}{c} w⊗y_{r_{1},m}>w⊗y_{r_{1},M}\\ \left(1-w\right)⊗y_{r_{2},m}>\left(1-w\right)⊗y_{r_{2},M} \end{array}\\ & \Rightarrow w⊗y_{r_{1},m}+\left(1-w\right)⊗y_{r_{2},m}>w⊗y_{r_{1},M}+\left(1-w\right)⊗y_{r_{2},M}\\ & \Rightarrow y_{m}^{\text{new}}>y_{M}^{\text{new}}. \end{array}$$

## Simulation study

### Data simulation

The simulated data are generated by using a multinomial logistic regression. The main idea is to create a probability distribution from a multinomial logistic regression, and then use a Dirichlet distribution with those probabilities to generate the actual label of the new point.

The notation is the same as in the previous section: the number of features (resp. classes) is *p* (resp. *K*), and the number of samples is *n*. The user has to specify a matrix *B* ∈ [0, 1]^(*p*+1)×*K*^ which corresponds to the regression coefficients, where *B*_*i*,*k*_ is associated with the *i*th feature and the *k*th class. For instance, for a class *k*, the regression coefficients will be (*B*_0,*k*_, *B*_1,*k*_, …, *B*_*p*,*k*_). Note that *B*_0,*k*_ is the intercept, hence explaining the (*p* + 1) × *K* dimension of *B*.

For a given point $x=\left(x_{1},\ldots ,x_{p}\right)\in R^{p}$, we define $x^{\prime }=\left(1,x_{1},\ldots ,x_{p}\right)\in R^{p+1}$ and a vector *α* as:

$$\begin{array}{cc} \alpha & =\text{softmax}(B_{0,1}+B_{1,1}x_{1}+\cdots +B_{p,1}x_{p},\ldots ,B_{0,K}+B_{1,K}x_{1}+\cdots +B_{p,K}x_{p})\\ & =\text{softmax}(x^{\prime }\cdot B_{\cdot ,1},\ldots ,x^{\prime }\cdot B_{\cdot ,K}). \end{array}$$

We are then able to randomly draw a label for *x* with a Dirichlet distribution with parameter *α*. Algorithm 3 generates a random dataset using this method.

To better understand how the regression coefficients *B* can change the configuration of the data, we give an example of simulated data with 2 features and 2 labels. Two different values *B*^(*a*)^ and *B*^(*b*)^ are tested:

$$B^{\left(a\right)}=[\begin{array}{cc} 0.4 & 0.4\\ 0.2 & 0.4\\ 0.5 & 0.3 \end{array}],\;B^{\left(b\right)}=[\begin{array}{cc} 0.1 & 0.9\\ 0.0 & 0.5\\ 0.8 & 0.1 \end{array}].$$

**Fig 3:**
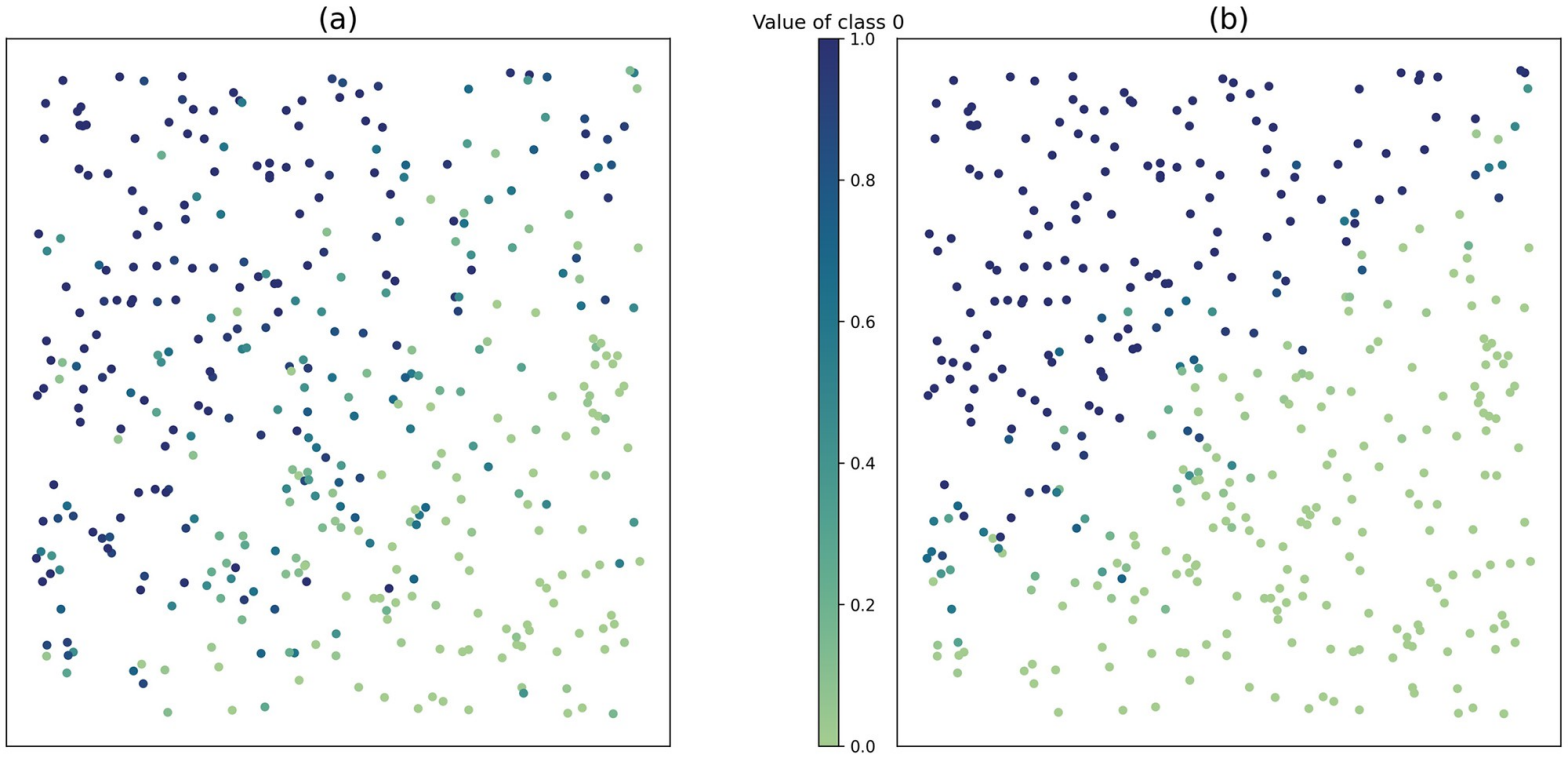
Simulation of 400 points using *B*^(*a*)^ (a) and *B*^(*b*)^ (b).

Each column of a matrix *B* represents the coefficients for one class. There are 3 lines here because there are 2 features and the first value corresponds to the intercept of the regression. In *B*^(*a*)^, the coefficients of each class are purposely close to each other, while they are easily separable in *B*^(*b*)^. Fig 3 shows the value of the labels when generating the same 400 points with each matrix, using the function *generate_dataset* of our *smote-cd* Python package, with *random_state* = 2. The points created with *B*^(*b*)^ have a clearer border between the points fully belonging in one class or the other. As there are only two classes and their sum is 1, it is only necessary to represent the value of one of them with the gradient of color.

**Algorithm 3** Function to generate a synthetic dataset with compositional labels

**Require**: K∈N the number of classes.

**Require**: p∈N the number of features.

**Require**: n∈N the number of samples.

**Require**: *B* ∈ [0, 1]^(*p*+1)×*K*^ the regression coefficients, where *B*_*m*,*k*_ is associated with the *m*th feature and the *k*th class.

**Ensure**: Generated data *X* ∈ *R*^*n*×*p*^ and *Y* ∈ *R*^*n*×*K*^

1: Create a random matrix of points *X* ∈ *R*^*n*×*p*^ such that for all *i*, *j*, *x*_*i*,*j*_ is a random number uniformly drawn in a chosen interval (for instance [-10, 10])

2: Initialize *Y* as an empty matrix of size (*n* × *K*).

3: **for** every row *x* in *X* (and its associated row index *i*) **do**

4:  Compute *α* = softmax(*x*′ ⋅ *B*_⋅,1_, …, *x*′ ⋅ *B*_⋅,*K*_) where *x*′ = (1, *x*_1_, *x*_2_, …, *x*_*p*_)

5:  Randomly draw a vector from a Dirichlet distribution with parameter *α* and attribute it to *y*_*i*,._, the *i*th row of *Y*.

6: **end for**

7: **return** *X*, *Y*

### Performance measures

The value of row *i* column *j* of *Y* is still denoted by *y*_*i*,*j*_, and is the probability that the *i*th sample belongs to class *j*. Let ${\hat{y}}_{i,j}$ be the estimate of this probability by a model.

Different metrics can be used to measure the performance of the model. A popular metric is the cross-entropy:

$$\begin{array}{c} CrossEntropy=-\frac{1}{n}\sum_{i=1}^{n}\sum_{j=1}^{K}y_{i,j}\;\text{log}\left({\hat{y}}_{i,j}+\varepsilon \right). \end{array}$$ (4)

The *ε* is added here to overcome the case where ${\hat{y}}_{i,j}=0$. We chose *ε* = 10^−20^. As the cross-entropy is a loss function, the smaller it is, the better the model performs. The cross-entropy loss may not always be suitable for our model because it treats each sample as equally important, without taking into account the imbalance of the test set. For instance, consider a model predicting three different classes (1, 2 and 3), and imagine that this model performs quite well on class 1 but poorly on classes 2 and 3. If the test set is imbalanced and has a large proportion of class 1 samples, the cross-entropy loss of this model will be low even though it performs poorly overall. The coefficient of determination *R*^2^ allows assessment of the performance of a model on each of the *K* classes. For a class *j*, the coefficient of determination is given by

$$\begin{array}{c} R_{j}^{2}=1-\frac{\sum_{i=1}^{n}{\left(y_{i,j}-{\hat{y}}_{i,j}\right)}^{2}}{\sum_{i=1}^{n}{\left(y_{i,j}-{\bar{y}}_{j}\right)}^{2}}, \end{array}$$

where ${\bar{y}}_{j}$ is the mean of the values of the *j*th class. The final *R*^2^ will be equal to the average of the $R_{j}^{2}$ for each class *j*.

In addition, we also use the Root Mean Squared Error (RMSE) to measure the accuracy of the models. Since we are dealing with multi-class compositional vectors, we define the RMSE between a true and estimated vector as the average of RMSEs calculated across all their classes. Specifically, this is calculated as:

$$\begin{array}{c} \text{RMSE}=\sqrt{\frac{1}{n}\frac{1}{K}\sum_{i=1}^{n}\sum_{j=1}^{K}{\left(y_{i,j}-{\hat{y}}_{i,j}\right)}^{2}}. \end{array}$$

Even though we are working on a regression problem, classification metrics can be a good tool to understand the efficiency of the models. To do so, it is easy to transform a compositional label *y*_*i*,⋅_ into a class $y_{i}^{\prime }$ by applying the argmax,

$$\begin{array}{c} y_{i}^{\prime }=\underset{j}{\text{argmax}\;}y_{i,j}. \end{array}$$

The usual classification metrics can then be applied to *y*′. Here, we will use the accuracy (the number of correct points divided by the total number of points) and the F1-score which is computed per class,

$$\begin{array}{c} \text{F1-score}\;=\frac{TP}{TP+\frac{1}{2}\left(FN+FP\right)}, \end{array}$$

where *TP* are the true positive, *FN* the false negative and *FP* the false positive. As with the *R*^2^, the F1-score will be computed for each class and then averaged.

### Results

**Fig 4:**
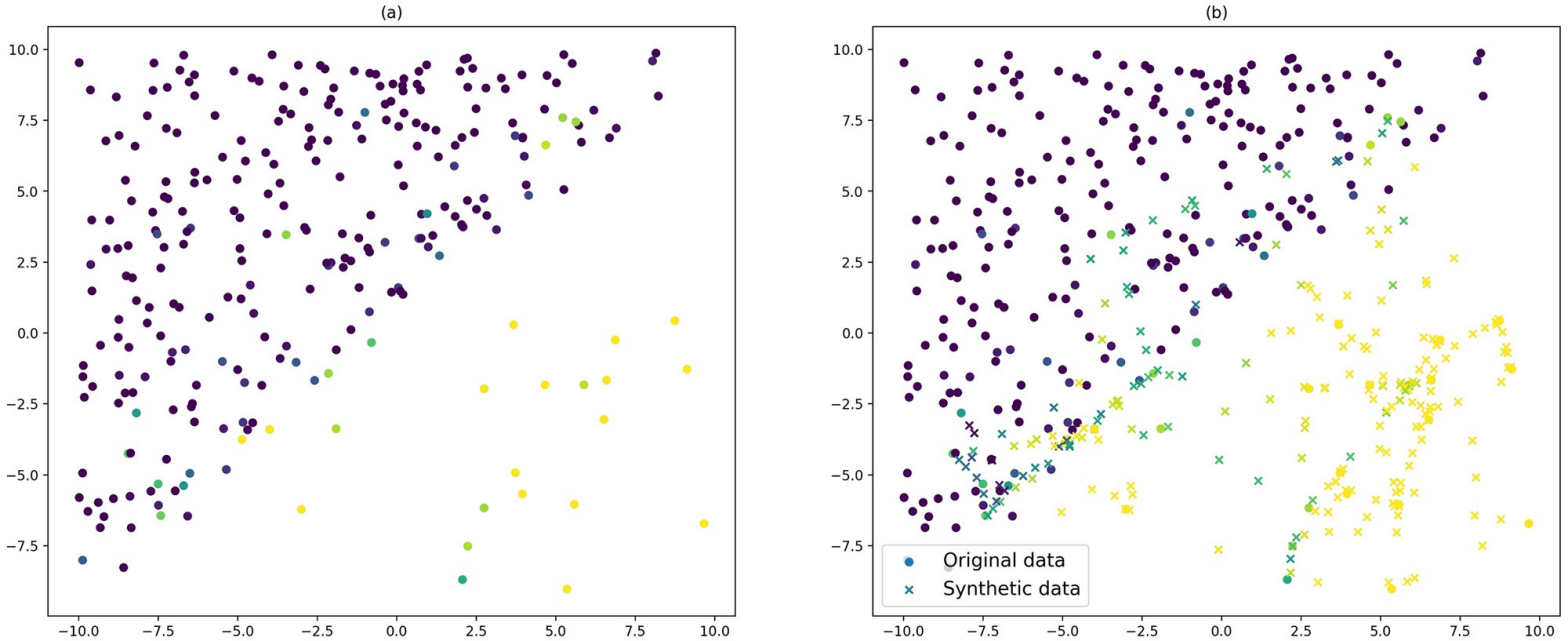
An example of SMOTE-CD. (a) The original imbalanced dataset, (b) the output balanced dataset with the created points displayed as a cross.

First, to investigate the effect of the oversampling technique, synthetic data were generated with 2 features and 2 classes. To make the dataset imbalanced, 90% of the points that had class 0 as a majority class were deleted. We obtain a dataset in which 93% of the points have class 1 as their majority class (Fig 4(a)), which is then oversampled by selecting a number of nearest neighbors *k* = 10. Fig 4(b) displays the balanced dataset after applying SMOTE-CD, where the original points are displayed as circles and the synthetic created points are displayed as crosses. As in Fig 3, the gradient of color represents the value of one of the two classes.

To evaluate the performance of SMOTE-CD, a 5-fold cross validation was used for three models: Gradient Boosting tree (GB), Neural Network (NN) with one hidden layer, and Dirichlet regression model [36]. The first and second models are chosen because Random Forest and NN are known to be the most efficient to map coral reefs from multispectral satellites [37, 38] and because NN are used in literature for the task of predicting compositional labels [39, 40], and the third is chosen because it is used to generate the simulated data. For each model, the performance is compared between the raw and oversampled data. For the models on which it is possible (GB and NN), hyperparameter tuning was been performed for each data (raw or oversampled). The hyperparameters are detailed in S1 and S2 Tables.

The simulated data were generated with the same shape as the Maupiti data. We selected a matrix *B* such that the imbalance of the classes was similar to the one of the real data (see Table 1). Then, 550 points were created with 16 features and 4 classes to train the models. Testing was performed with 11000 points (20 times the training set size). This operation was repeated 100 times with the same *B*. The results and metrics (accuracy, cross-entropy, average F1, RMSE and *R*^2^) are presented in Table 2.

**Table 2:** Comparison of simulated raw data (4 classes) and oversampled data, repeated 100 times. Displayed results are mean (s.d.).

	Accuracy	Cross-entropy	F1-score	RMSE	*R* ^2^
GB (raw)	0.692 (0.018)	5.272 (1.539)	0.532 (0.045)	0.363 (0.011)	0.137 (0.067)
GB (logratio)	0.724 (0.017)	2.508 (0.553)	0.658 (0.027)	0.341 (0.011)	0.198 (0.074)
GB (compositional)	0.683 (0.016)	3.657 (1.055)	0.604 (0.038)	0.359 (0.011)	0.139 (0.085)
NN (raw)	0.772 (0.026)	3.340 (1.370)	0.611 (0.057)	0.315 (0.020)	0.298 (0.103)
NN (logratio)	0.784 (0.023)	1.700 (0.380)	0.729 (0.033)	0.304 (0.018)	0.301 (0.108)
NN (compositional)	0.750 (0.054)	3.483 (1.367)	0.690 (0.063)	0.332 (0.040)	0.198 (0.207)
Dirichlet (raw)	0.789 (0.016)	0.685 (0.017)	0.605 (0.039)	0.287 (0.004)	0.416 (0.022)
Dirichlet (logratio)	0.875 (0.010)	0.754 (0.017)	0.824 (0.019)	0.303 (0.004)	0.380 (0.022)
Dirichlet (compositional)	0.874 (0.011)	0.755 (0.017)	0.824 (0.019)	0.303 (0.004)	0.379 (0.022)

For both the Gradient Boosting and Neural Network models, the oversampling with logratio distance significantly improves all metrics except for *R*^2^ on the Neural Network (p < 0.0006). With the compositional distance on the Neural Network, only the F1-score significantly increases (p ≪ 10^−10^), while accuracy, RMSE, and *R*^2^ decrease. The GB model shows significant improvement for cross-entropy, F1-score, and RMSE (p < 0.008), but a decrease in accuracy. The Dirichlet model with oversampling significantly increases accuracy and F1-score (p ≪ 10^−10^) but decreases cross-entropy, RMSE, and *R*^2^.

**Fig 5:**
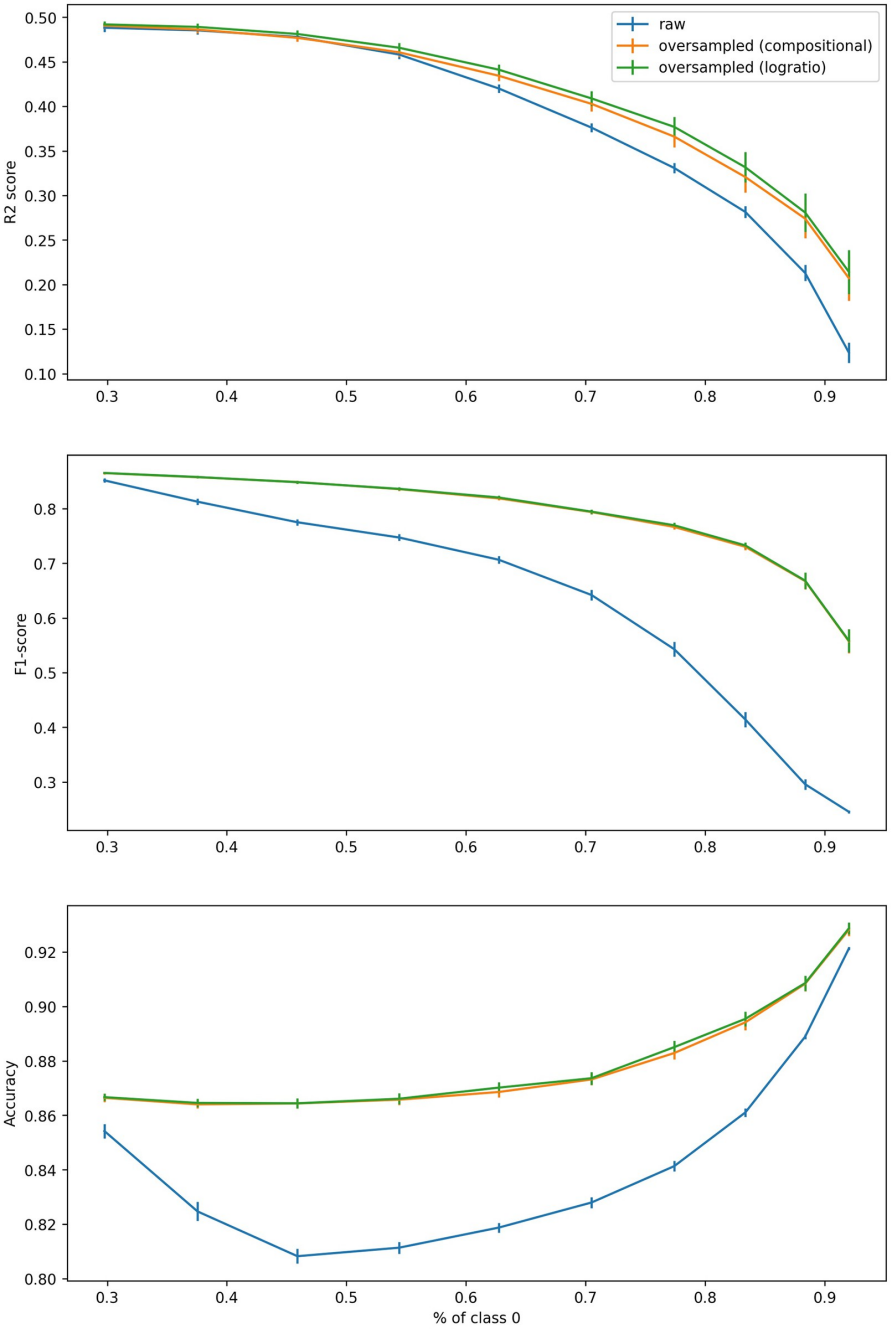
Performance of Dirichlet model on raw and oversampled data, depending on the imbalance of the dataset (indicated by % of observations in class 0), based on 16 features and 4 classes.

In order to understand the effects of the imbalance of the dataset on the performance of the oversampling method, three metrics (accuracy, F1 and *R*^2^) were evaluated with different imbalance ratios. First, a matrix *B* was created to generate a balanced dataset with 16 features and 4 classes. Then, the ratio of class 0 was increased by incrementing the value of *B*_1,1_. At each step (for a total of ten steps), the following operation was repeated 100 times: 550 points were created to train the models on the raw or oversampled data, and the models were tested on a set of 11000 points. The result appears in Fig 5.

It is apparent that the efficiency of SMOTE-CD depends on the data and the model used. The oversampling technique only improves the *R*^2^ score when the dataset is slightly imbalanced (largest class representing less than 40%), but performs poorly when it is highly imbalanced. On the other hand, the more the dataset is imbalanced, the more the oversampling technique will improve the F1-score. The improvement in accuracy peaks at a certain value of imbalance (when the largest class represents 50% of the dataset), but drops above that threshold.

**Fig 6:**
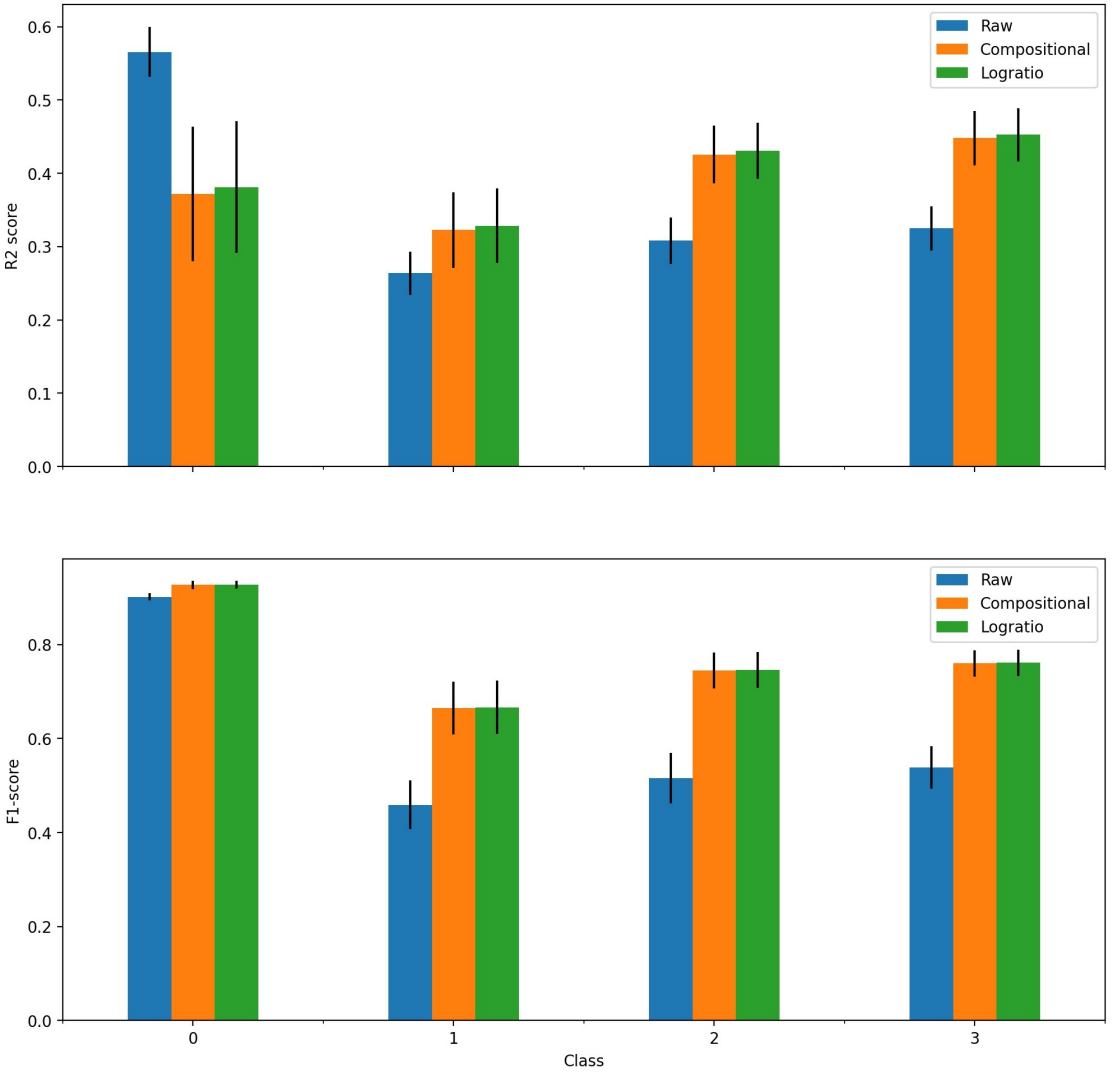
Average *R*^2^ and F1-score per class of Dirichlet model on raw and oversampled simulated data. Bars represent the mean score, vertical lines represent the standard deviation.

In order to explain the low *R*^2^ score for the oversampled data, the *R*^2^ per class was calculated for each of the ten steps mentioned above and then averaged. Fig 6 displays the result. The average imbalance ratio is 52% for class 0 (and thus approximately 16% for the three other classes).

For the largest class, the *R*^2^ score is decreased from 0.5 to 0.3 by the oversampling technique, which explains why the raw score is higher than the oversampled score in Fig 5. However, for the three minority classes, the *R*^2^ is increased by approximately 0.05, which is the initial goal of the method.

Similarly, Fig 6 also depicts the F1-score per class, averaged over the seven steps. The difference is that the F1-score of the majority class is not decreased by the oversampling technique, while the score of the minority classes is increased by approximately 0.08.

## Application to Maupiti data

The performance of the three models on the raw dataset was compared with the oversampled dataset (with either the logratio distance used to create the new labels, or the compositional distance). The results are shown in Table 3. With the Maupiti dataset, the NN is defined with 2 hidden layers of size 80 and 40, and the relu activation function.

**Table 3:** Results comparing raw Maupiti data (4 classes) and oversampled with a 5-fold cross validation. Displayed results are mean (s.d.).

	Accuracy	Cross-entropy	F1-score	RMSE	*R* ^2^
GB (raw)	0.857 (0.003)	2.538 (0.196)	0.809 (0.031)	0.229 (0.003)	0.583 (0.018)
GB (logratio)	0.859 (0.003)	2.504 (0.182)	0.822 (0.028)	0.226 (0.003)	0.596 (0.019)
GB (compositional)	0.859 (0.004)	2.486 (0.149)	0.822 (0.028)	0.226 (0.003)	0.596 (0.018)
NN (raw)	0.877 (0.003)	4.048 (0.416)	0.831 (0.008)	0.214 (0.003)	0.624 (0.018)
NN (logratio)	0.877 (0.003)	3.982 (0.456)	0.835 (0.009)	0.214 (0.003)	0.623 (0.017)
NN (compositional)	0.878 (0.003)	3.956 (0.406)	0.834 (0.010)	0.213 (0.003)	0.622 (0.020)
Dirichlet (raw)	0.801 (0.056)	1.676 (0.874)	0.684 (0.127)	0.262 (0.033)	0.420 (0.163)
Dirichlet (logratio)	0.810 (0.049)	1.663 (0.851)	0.762 (0.064)	0.262 (0.036)	0.423 (0.174)
Dirichlet (compositional)	0.810 (0.049)	1.654 (0.839)	0.762 (0.064)	0.262 (0.036)	0.423 (0.174)

With the GB model, all the metrics are significantly improved (p < 0.03) when using the oversampling technique, excepted for the cross-entropy for which the differences are not statistically significant (p = 0.14 and p = 0.38 respectively for the compositional and the logratio distance). The SMOTE-CD shows less results with the NN and Dirichlet model, where only the difference on the F1 is statistically significant (respectively p < 0.044) and p < 10^−10^). This improvement is quite important for the Dirichlet model though, as it represents a difference of almost 0.08.

**Fig 7:**
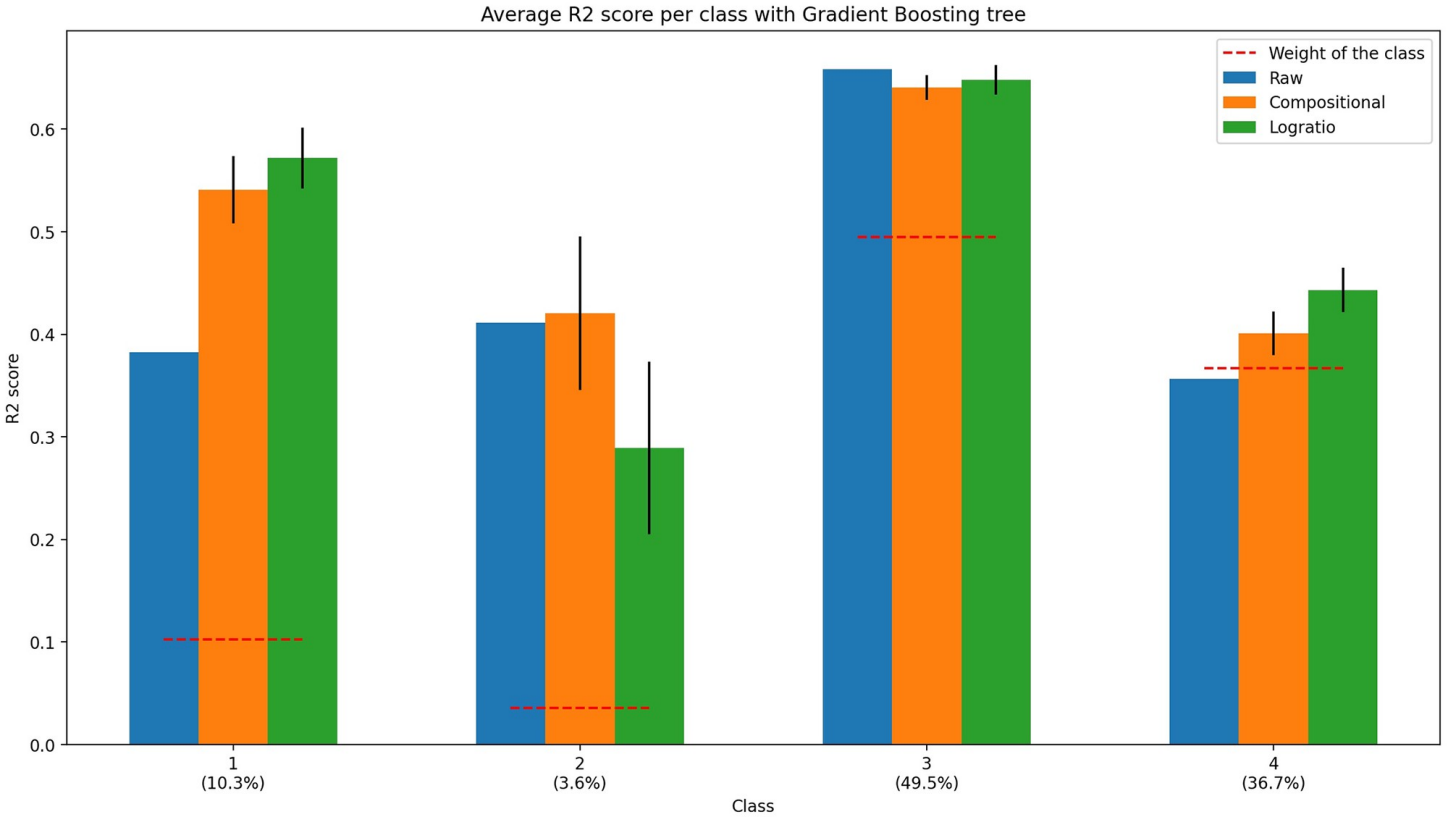
Average *R*^2^ score per class of Gradient Boosting tree on raw and oversampled Maupiti data. The red dotted lines represent the weight of each class, and the value below the class is its weight. Bars represent the mean score, vertical lines represent the standard deviation.

We analyze the per-class *R*^2^ of the Gradient Boosting tree as it is the best model. Fig 7 compares the *R*^2^ between the raw and oversampled data. The oversampling technique decreases the performance of the model for the smallest class (Class 2) for the logratio distance, does not change for the largest class (Class 3) and increases the performance on the others (Classes 1 and 4).

We conclude that SMOTE-CD does not improve the performance for a class that is too small: in order to perform ideally, it requires enough points to oversample.

## Application to Tecator dataset

To fully evaluate the effectiveness of the SMOTE-CD technique, we applied it to the Tecator meat sample dataset [41], which consists of 240 meat samples. Each sample has absorbance values measured at 100 different wavelengths, as well as corresponding information on the composition of moisture (water), fat, and protein contents. The objective of this analysis is to predict a 3-class compositional data vector from a feature vector of size 100. Because the Dirichlet regression model can be very slow when dealing with a high number of features, we opted to improve its speed by using only the 22 principal components provided in the dataset instead of the 100 features.

To account for the small size of the dataset, a 10-fold cross validation is applied for each model, iterated over 100 times to vary the folds. The results are displayed in Table 4. The neural network is configured with three hidden layers, each having 70 neurons and using the hyperbolic tangent (tanh) activation function, which were selected through hyperparameter tuning.

**Table 4:** Results comparing raw Tecator data (3 classes) and oversampled with a 10-fold cross validation, iterated 100 times. Displayed results are mean (s.d.).

Model	Accuracy	Cross-entropy	F1-score	RMSE	*R* ^2^
GB (raw)	0.932 (0.006)	0.860 (0.001)	0.701 (0.042)	0.046 (0.001)	0.717 (0.023)
GB (logratio)	0.957 (0.008)	0.860 (0.001)	0.830 (0.046)	0.044 (0.002)	0.730 (0.027)
GB (compositional)	0.957 (0.008)	0.860 (0.002)	0.834 (0.046)	0.044 (0.002)	0.730 (0.026)
NN (raw)	0.908 (0.000)	0.928 (0.009)	0.512 (0.036)	0.113 (0.005)	-1.230 (0.484)
NN (logratio)	0.904 (0.014)	0.938 (0.010)	0.513 (0.044)	0.122 (0.007)	-1.156 (0.449)
NN (compositional)	0.904 (0.016)	0.937 (0.010)	0.512 (0.044)	0.122 (0.007)	-1.158 (0.466)
Dirichlet (raw)	0.954 (0.007)	0.852 (0.003)	0.846 (0.037)	0.048 (0.003)	0.708 (0.045)
Dirichlet (logratio)	0.940 (0.011)	0.878 (0.006)	0.800 (0.044)	0.072 (0.006)	0.310 (0.224)
Dirichlet (compositional)	0.940 (0.011)	0.877 (0.005)	0.802 (0.037)	0.072 (0.005)	0.323 (0.413)

With the NN, the raw data gives slightly better performances than the oversampled data. However, given the really poor performances of the NN (a negative *R*^2^ and a really high RMSE), we also note that this model was probably not suited for this dataset.

The analysis of the GB and Dirichlet models reveals interesting differences. In both cases, using either the raw or oversampled datasets leads to statistically significant differences (p < 10^−4^). Specifically, for the GB model, using the oversampled data results in better performance, while for the Dirichlet model, oversampling decreases the performance. Notably, among all the models tested, the GB model trained on oversampled data with compositional distance yields the best results. Compared to the Dirichlet model trained on raw data, this approach achieves significantly better accuracy (p < 0.006), RMSE (p ≪ 10^−10^), and *R*^2^ (p < 10^−4^), with only a slight difference of 1% in cross-entropy and F1-score.

In light of these results, it is apparent that SMOTE-CD can improve the performance for a model that does not perform too poorly (e.g. a *R*^2^ above 0.3). Indeed, if a model has low performance, it is more likely that this is due to poor fit to the data than from the imbalance of the dataset.

## Discussion

The results on the synthetic datasets show that the SMOTE-CD technique can significantly improve the F1-score and accuracy, but it has a mixed effect on other metrics depending on the model and dataset imbalance level. SMOTE-CD improves the overall performance of the model, especially with respect to the accuracy and the F1-score in the cases where the dataset is not too heavily imbalanced. The *R*^2^ score of the majority class remains similar, but the *R*^2^ of a very small class (3% of the dataset) will be decreased. The *R*^2^ of all the other classes is improved, which is the desired goal of the method.

The results on the real datasets show that the SMOTE-CD technique can significantly improve the performance of the Gradient Boosting model for all metrics, while it has a less pronounced effect on the other models. The per-class analysis of the *R*^2^ score reveals that the SMOTE-CD technique can improve the performance for some classes but not for others, depending on the model and distance metric used.

Further tests are required with other datasets having compositional labels, but these are often hard to find because they are not publicly available. Our oversampling technique could be used with datasets in biology and metabolomics, in poll studies or in soil analysis, but its effectiveness depends on several factors that should be carefully considered.

The original SMOTE paper [3] proposes to undersample the dataset before applying the oversampling technique, which we similarly tested here. The synthetic dataset was first undersampled by randomly withdrawing some points from the majority class, until the total sum of the largest class was equal to the sum of the second largest one. SMOTE-CD was then applied. The results are summarised in S3 Table and compared with those in Table 2 when not using undersampling (S4 Table). No significant difference can be seen when using undersampling before the oversampling, be it positive or negative. The results are similar when undersampling not only the points having the largest class as their majority class, but the points having one of the *n* largest classes as their majority class (with *n* ∈ [1, …, 3]). At this point, we are not able to exclude the utility of the undersampling and suggest it could once more depend on the dataset or on the way the removed points are chosen. For instance, when performing random undersampling, consideration could be given to an Edited Nearest Neighbor approach [42]; see [43].

Work has still to be done regarding the initial selection of the points, because it can influence the performance of the original SMOTE algorithm. For instance, we could imagine attributing a “safe” level to each point by exploring its *k* nearest neighbors and using it in the creation of a new point [44]. It would also be possible to only oversample the points on the border [45], where the border would here be defined by the points having a given amount of neighbors that have the largest class as their majority class.

## Conclusion

The SMOTE algorithm has been adapted to deal with the special case in which the dataset labels are compositional, which had not been done before. The present study investigates its effectiveness on imbalanced datasets for three different models: Gradient Boosting tree, Neural Networks, and Dirichlet Regression. The evaluation was performed on both synthetic and real datasets, and several metrics, including accuracy, F1-score, RMSE, cross-entropy, and *R*^2^, were used to assess the performance of the models.

The study suggests that the effectiveness of the SMOTE-CD technique depends on several factors, including the model, distance metric, dataset imbalance level, and class distribution. The SMOTE-CD technique can improve the performance of a model that does not perform too poorly, but it may not be effective for a model with very low performance.

An implementation is proposed in the Python package *smote-cd* available on PyPi: https://pypi.org/project/smote-cd. The Jupyter notebooks used to simulate the data and perform the analyses can be found on the GitHub page of the package: https://github.com/teongu/smote_cd.

## Supporting information

S1 Table Hyperparameters of the Gradient Boosting tree. The hyperparameters listed here are those applied to the Gradient Boosting tree of the Python package *scikit-learn*, tuned with the *hyperopt* package. The value of the *random_state* is 2.
(PDF)

S2 Table Hyperparameters of the Neural Networks. The hyperparameters listed here are those applied to the MLPRegressor of the Python package *scikit-learn*, tuned with the *hyperopt* package. The value of the *random_state* is 2.
(PDF)

S3 Table Results comparing simulated raw data (4 classes) and oversampled repeated 100 times, when applying undersampling beforehand. (PDF)

S4 Table Difference when applying undersampling+oversampling, and oversampling only. Results are in bold when the undersampling provides better results.
(PDF)
