# Supplementary material for: SMOTE-CD: SMOTE for compositional data
Source: PLoS One. 2023 Jun 29;18(6):e0287705. doi: 10.1371/journal.pone.0287705 (PMC10309641; doi:10.1371/journal.pone.0287705)
Supplement: S1 Table — The hyperparameters listed here are those applied to the Gradient Boosting tree of the Python package scikit-learn, tuned with the hyperopt package. The value of the random_state is 2. (PDF) [file pone.0287705.s001.pdf]

Supporting information: S1 Table

Table 1. Hyperparameters of the Gradient Boosting tree. The hyperparameters listed here are those applied to the Gradient Boosting tree of the Python package *scikit-learn*, tuned with the *hyperopt* package. The value of the *random_state* is 2.

	Raw	Oversampled (compositional)	Oversampled (logratio)
ccp_alpha	10	10	0.5
learning_rate	0.01	0.01	0.01
max_depth	5	5	4
max_features	log2	sqrt	sqrt
min_samples_leaf	10	1	10
n_estimators	200	100	200
