# Supplementary material for: SMOTE-CD: SMOTE for compositional data
Source: PLoS One. 2023 Jun 29;18(6):e0287705. doi: 10.1371/journal.pone.0287705 (PMC10309641; doi:10.1371/journal.pone.0287705)
Supplement: S2 Table — The hyperparameters listed here are those applied to the MLPRegressor of the Python package scikit-learn, tuned with the hyperopt package. The value of the random_state is 2. (PDF) [file pone.0287705.s002.pdf]

Supporting information: S2 Table

Table 2. Hyperparameters of the Neural Networks. The hyperparameters listed here are those applied to the MLPRegressor of the Python package *scikit-learn*, tuned with the *hyperopt* package. The value of the *random_state* is 2.

	Raw	Oversampled (compositional)	Oversampled (logratio)
activation	identity	logistic	identity
alpha	$1e^{-5}$	$1e^{-3}$	$1e^{-3}$
beta_1	0.95	0.95	0.9
hidden_layer_sizes	(40,)	(20,)	(80,)
learning_rate	constant	constant	constant
learning_rate_init	0.001	0.0001	0.0001
max_iter	10000	10000	10000
momentum	0.9	0.8	0.9
solver	sgd	adam	adam
