# Supplementary material for: SMOTE-CD: SMOTE for compositional data
Source: PLoS One. 2023 Jun 29;18(6):e0287705. doi: 10.1371/journal.pone.0287705 (PMC10309641; doi:10.1371/journal.pone.0287705)
Supplement: S3 Table — (PDF) [file pone.0287705.s003.pdf]

# Supporting information: S3 Table

**Table 3. Results comparing simulated raw data (4 classes) and oversampled repeated 100 times, when applying undersampling beforehand.**

| | $R^2$ | Accuracy | F1-score |
|---|---|---|---|
| GB (raw) | 0.141 (0.194) | 0.694 (0.061) | 0.526 (0.138) |
| GB (logratio) | 0.147 (0.214) | 0.707 (0.036) | 0.635 (0.087) |
| GB (compositional) | 0.130 (0.255) | 0.688 (0.036) | 0.600 (0.087) |
| NN (raw) | 0.302 (0.306) | 0.773 (0.816) | 0.610 (0.173) |
| NN (logratio) | 0.295 (0.311) | 0.784 (0.046) | 0.727 (0.092) |
| NN (compositional) | 0.212 (0.668) | 0.754 (0.158) | 0.694 (0.189) |
| Dirichlet (raw) | 0.413 (0.056) | 0.781 (0.051) | 0.594 (0.102) |
| Dirichlet (logratio) | 0.379 (0.066) | 0.874 (0.031) | 0.823 (0.056) |
| Dirichlet (compositional) | 0.381 (0.071) | 0.874 (0.026) | 0.824 (0.056) |
