# Supplementary material for: SMOTE-CD: SMOTE for compositional data
Source: PLoS One. 2023 Jun 29;18(6):e0287705. doi: 10.1371/journal.pone.0287705 (PMC10309641; doi:10.1371/journal.pone.0287705)
Supplement: S4 Table — Results are in bold when the undersampling provides better results. (PDF) [file pone.0287705.s004.pdf]

Supporting information: S4 Table

Table 4. Difference when applying undersampling+oversampling, and oversampling only. Results are in bold when the undersampling provides better results.

	R^2	Accuracy	F1-score
GB (logratio)	-0.05	-0.017	-0.022
GB (compositional)	-0.009	**0.006**	-0.003
NN (logratio)	-0.008	0	-0.003
NN (compositional)	**0.019**	**0.006**	**0.005**
Dirichlet (logratio)	-0.002	-0.001	-0.002
Dirichlet (compositional)	**0.002**	0	**0.001**
